# Advanced 4-dimensional cone-beam computed tomography reconstruction by combining motion estimation, motion-compensated reconstruction, biomechanical modeling and deep learning

**DOI:** 10.1186/s42492-019-0033-6

**Published:** 2019-12-12

**Authors:** You Zhang, Xiaokun Huang, Jing Wang

**Affiliations:** Division of Medical Physics and Engineering, Department of Radiation Oncology, UT Southwestern Medical Center, 2280 Inwood Road, Dallas, TX 75390 USA

**Keywords:** Cone-beam computed tomography, Image reconstruction, Motion estimation, Biomechanical modeling, Deep learning

## Abstract

4-Dimensional cone-beam computed tomography (4D-CBCT) offers several key advantages over conventional 3D-CBCT in moving target localization/delineation, structure de-blurring, target motion tracking, treatment dose accumulation and adaptive radiation therapy. However, the use of the 4D-CBCT in current radiation therapy practices has been limited, mostly due to its sub-optimal image quality from limited angular sampling of cone-beam projections. In this study, we summarized the recent developments of 4D-CBCT reconstruction techniques for image quality improvement, and introduced our developments of a new 4D-CBCT reconstruction technique which features simultaneous motion estimation and image reconstruction (SMEIR). Based on the original SMEIR scheme, biomechanical modeling-guided SMEIR (SMEIR-Bio) was introduced to further improve the reconstruction accuracy of fine details in lung 4D-CBCTs. To improve the efficiency of reconstruction, we recently developed a U-net-based deformation-vector-field (DVF) optimization technique to leverage a population-based deep learning scheme to improve the accuracy of intra-lung DVFs (SMEIR-Unet), without explicit biomechanical modeling. Details of each of the SMEIR, SMEIR-Bio and SMEIR-Unet techniques were included in this study, along with the corresponding results comparing the reconstruction accuracy in terms of CBCT images and the DVFs. We also discussed the application prospects of the SMEIR-type techniques in image-guided radiation therapy and adaptive radiation therapy, and presented potential schemes on future developments to achieve faster and more accurate 4D-CBCT imaging.

## Introduction

Accurate tumor/target localization is key to safe, precise and effective radiotherapy [1]. Cone-beam computed tomography (CBCT) imaging has become a standard-of-care in a majority of the radiotherapy clinics, with its successful capture of volumetric anatomical information to guide accurate on-board target localization and setup correction [2, 3]. In principal, the 3-dimensional CBCT (3D-CBCT) technique acquires 2D cone-beam projections from varying beam angles, usually covering an angle span of at least 200° [4], to reconstruct 3D volumetric information. Compared with 2D projections, the overlaying effects have been removed by 3D-CBCT to allow better soft-tissue contrast and more accurate structure localization in 3D [2]. However, there are multiple remaining issues with the current CBCT technique. One major issue is the motion-induced imaging artifacts and blurring for motion-involved sites such as lung and liver, which may severely reduce the tumor localization accuracy [5, 6]. The conventional 3D-CBCT technique acquires cone-beam projections and reconstructs them into a single CBCT volume without considering the motion of anatomical structures across the projections. Due to the position variations of anatomical structures, combining all projections into one reconstruction will lead to motion blurriness and inaccurate target localization. To effectively suppress the motion blurriness, methods have been proposed to develop the respiratory-correlated CBCT, also known as 4-dimensional CBCT (4D-CBCT), to capture the motion in the fourth dimension (in addition to the three spatial dimensions) [7–9]. The key idea of 4D-CBCT is phase-sorting [10], which sub-groups the cone-beam projections into different respiratory phase bins according to their relative positioning on a nominal respiratory cycle. The relative positioning can be determined via an external landmark, such as the real-time position management system or the Anzai Belt [11, 12]; or via an internal landmark, such as the diaphragm position or the fiducial marker position [7]. More advanced signals based on Fourier transform have also been investigated to be successful [10]. Based on these positioning signals, projections were sorted into different bins. Within each phase bin, the projections are semi-static with minimal intra-phase motion. CBCTs reconstructed from these phase-sorted projections will have the motion blurriness subdued, leading to more accurate delineation and localization of anatomical structures. Stacking these phase-specific CBCT images together will also reveal the full motion trajectory of anatomical structures to guide tumor targeting and organ-of-risk sparing. Though conceptually concise and convenient to implement, the use of 4D-CBCT in clinics is currently limited. One major concern is that the phase-sorting process employed by 4D-CBCT leads to insufficient projections inside each phase bin (angular under-sampling), causing severe imaging streaking artifacts to the clinical CBCT reconstruction technique, the Feldkamp-Davis-Kress (FDK) algorithm [13, 14].

One straightforward solution is to acquire more projections to achieve sufficient angular sampling even after phase-sorting [4]. Such a strategy, however, is not practical due to the accompanying excessive imaging dose, which may induce secondary cancers [15]. Acquiring more projections also prolongs the imaging and treatment time, which may increase patient on-board position deviations [16], and add additional logistic burdens to the clinics. Many groups have tried to develop new reconstruction techniques, mostly iterative in nature, to improve the 4D-CBCT image quality from under-sampled acquisition. Many of these techniques rely on regularization techniques via metrics like total variation [17, 18], non-local means [19, 20] or wavelet frames [21] to explore the data sparsity and reduce the noises/artifacts of under-sampling. Substantial image quality improvement has been observed, and quantitatively validated via metrics like signal-to-noise ratio, root-mean-squared-error (RMSE) or universal quality index (UQI) [22]. However, these techniques are susceptible to displace or smooth out fine details and low-contrast anatomies from the reconstructed images.

Another type of CBCT reconstruction technique tries to incorporate prior information into the reconstruction process, such as the 2D-3D deformation method [23–28]. Instead of directly reconstructing CBCT images from acquired projections, the 2D-3D deformation method views the new CBCT volume as a deformation of prior CT/CBCT images, and translates the CBCT reconstruction into a deformation-vector-field (DVF) optimization problem. For each to-be-deformed CT/CBCT image, the DVFs usually compose of three matrices in 3D, each matrix with the same dimension as the to-be-deformed CT/CBCT image. The three matrices indicate the deformation along the three Cartesian directions, x, y and z, respectively [25]. The 2D-3D deformation algorithm iteratively optimizes the DVF, such that it deforms the prior image until the digitally-reconstructed-radiographs (DRRs) of the deformed image match the acquired cone-beam projections. The 2D-3D deformation technique can not only generate 4D-CBCT images, but provide DVFs to potentially allow automatic tumor localization, target tracking, dose accumulation and adaptive radiation therapy [29–32]. The incorporation of high-quality prior information also introduces more accurate Hounsfield units [30], and allows further imaging dose reduction by acquiring fewer projections for reconstruction. However, this technique cannot reconstruct non-deformation-induced intensity changes in new CBCT volumes, since the new CBCT is simplified as a purely deformed volume of the prior image [33].

In addition to these two types of techniques, motion-compensated reconstruction is another reconstruction technique to address the 4D-CBCT under-sampling issue [34–37]. The motion-compensation technique reconstructs a reference phase CBCT image through an estimated inter-phase motion model [34]. The motion model relates other phases of the 4D-CBCT to the reference phase in the form of inter-phase DVFs. Through applying the inter-phase DVFs, motion-compensated reconstruction can combine projections from all phases to achieve sufficient angular sampling. After the reference phase is reconstructed, the other phases can be derived using corresponding inverse DVFs. Unfortunately, the motion-compensation technique is limited by the inter-phase motion model accuracy, since a prior-information-driven motion model can be invalidated after motion pattern changes [25, 34, 36]. To have the model up-to-date, studies have proposed to estimate an on-board motion model directly between 4D-CBCT phase images reconstructed from under-sampled projections [35, 37]. However, the accuracy of these on-board motion models can still be impaired by the artifacts presented in the low-quality CBCT phase images.

In response to the current challenges of high-quality 4D-CBCT imaging, we have developed a simultaneous motion estimation and image reconstruction (SMEIR) technique [38–40], which combines total variation-based image regularization, 2D-3D deformation-driven motion model estimation, and motion-compensated reconstruction into a comprehensive reconstruction scheme. In comparison to the previous techniques, the SMEIR algorithm estimates an inter-phase motion model via 2D-3D deformation from a motion-compensated CBCT (mCBCT) and the phase-specific projections. The improved motion model is subsequently fed back into the motion-compensated reconstruction to update the mCBCT. Total variation-regularization is incorporated into the motion-compensated reconstruction to further improve the CBCT image quality. The resulting mCBCT, with improved accuracy and quality, is iteratively fed back into the 2D-3D deformation to dynamically update the motion model. The 2D-3D motion estimation and the motion-compensated image reconstruction form an iterative loop to continuously update the mCBCT image as well as the motion model until final convergence. The 4D-CBCT images at other phases are deformed from the mCBCT via the inverse DVFs simultaneously-optimized by the 2D-3D deformation algorithm. In this study, we detailed the general philosophy and workflow of the SMEIR algorithm, and introduced two new developments based on the original SMEIR algorithm: the biomechanical modeling-guided SMEIR (SMERI-Bio) and the SMEIR algorithm with artificial intelligence (AI)-driven DVF fine-tuning (SMEIR-Unet) [41]. In comparison to the original SMEIR algorithm, the SMEIR-Bio algorithm introduced biomechanical modeling to improve the intra-lung DVF accuracy to better reconstruct the fine details in lung, and better capture their motion. Similar to SMEIR-Bio, the SMEIR-Unet algorithm was developed to fine-tune intra-lung DVFs, however via a deep learning-driven approach. Compared with SMEIR-Bio, SMEIR-Unet may allow improved efficiency with reduced complexity and computational load. Corresponding reconstruction results of both digital anthropomorphic phantoms and real lung patient data were presented to illustrate the strengths and weaknesses of different methods. In the end, a review was further included to discuss the application prospects of the developed algorithms, specifically in terms of real-time image-guided radiotherapy and adaptive radiotherapy.

## Methods

### The original SMEIR algorithm

**Fig. 1:**
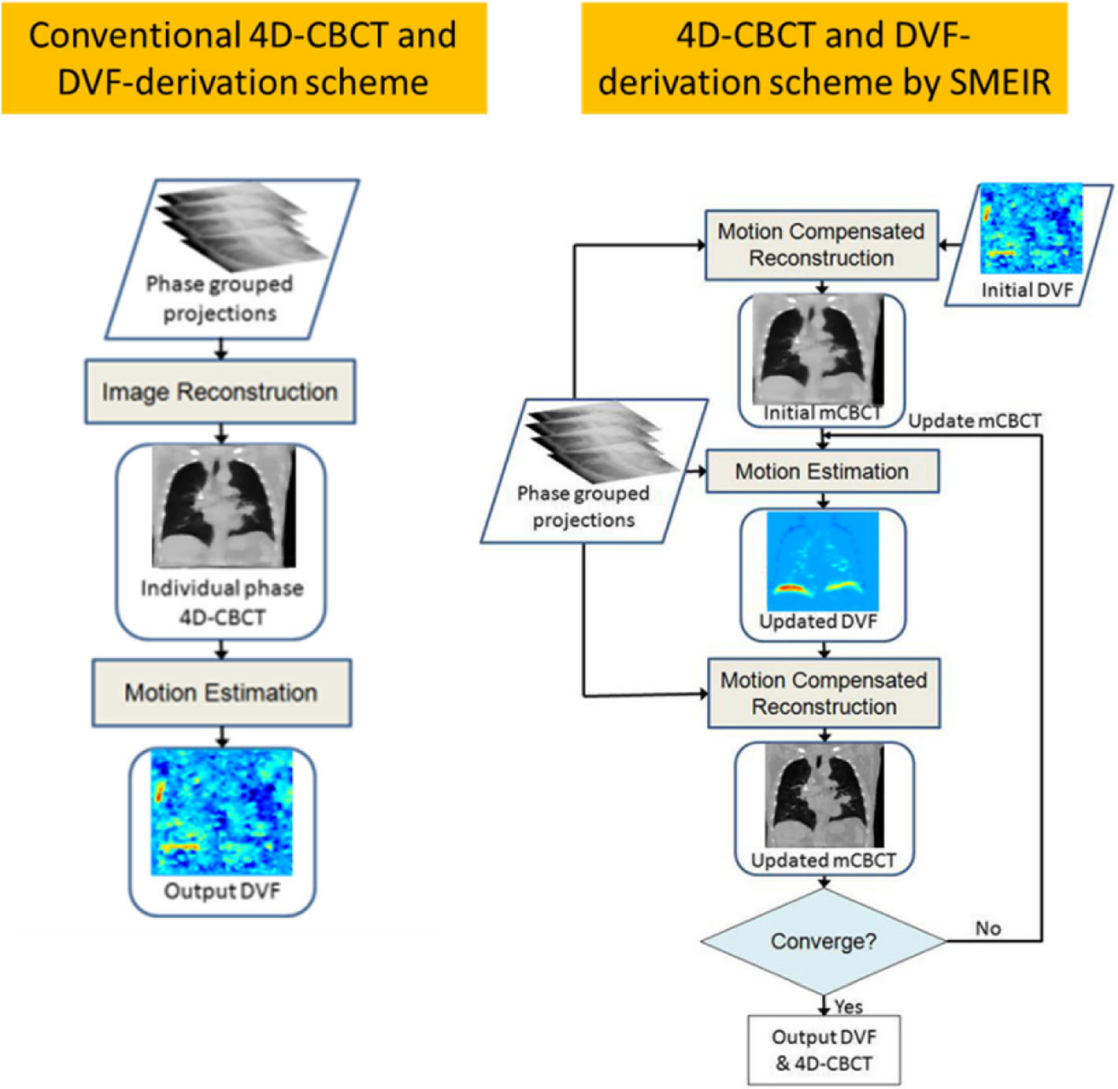
Flow-chart comparison between (a) the conventional 4D-CBCT reconstruction and DVF-derivation scheme for image-guided radiation therapy/adaptive radiation therapy applications, and (b) the proposed SMEIR scheme. Compared with the conventional scheme, the SMEIR scheme updates the 4D-CBCT images and the DVFs simultaneously and iteratively in an interleaved fashion. By SMEIR, the mCBCT is updated towards improved quality to drive more accurate DVF solution, and the more accurate DVFs subsequently improve the accuracy of the motion-compensated reconstruction for mCBCT. 4D-CBCT: 4-Dimensional cone-beam computed tomography; SMEIR: Simultaneous motion estimation and image reconstruction; DVF: Deformation-vector-field; mCBCT: Motion-compensated CBCT

The SMEIR algorithm integrates the motion estimation and image reconstruction to solve both the 4D-CBCT images and the corresponding inter-phase DVFs simultaneously. Compared with the conventional sequential scheme (Fig. 1), both the 4D-CBCT images and the DVFs are iteratively and dynamically updated by SMEIR to maximize the sharing of information to improve image and DVF accuracy.

The SMEIR algorithm is comprised of two major components, the motion estimation and the motion-compensated reconstruction. For motion estimation, the 2D-3D deformation technique is used, which is based on optimizing objective functions as shown in Eqs. 1 and 2:

1 $$ {f}_1\left({\mathrm{DVF}}^{0\to t}\right)={\left\Vert {p}^t-A{\mu}^0\left(x+{\mathrm{DVF}}^{0\to t}\right)\right\Vert}_{l_2}^2+\beta \ast E\left({\mathrm{DVF}}^{0\to t}\right) $$

2 $$ {f}_2\left({\mathrm{DVF}}^{t\to 0}\right)={\left\Vert {p}^0-A{\mu}^t\left(x+{\mathrm{DVF}}^{t\to 0}\right)\right\Vert}_{l_2}^2+\beta \ast E\left({\mathrm{DVF}}^{t\to 0}\right) $$

*p* denotes the 2D, phase-binned cone-beam projections, with the superscript indicating the respiratory phase they belong to; “ *A* ” denotes the projecting matrix of the projections *p*, which generates DRRs corresponding to the projections; “ 0 ” indicates the reference phase (motion-compensated phase); “ *t* ” denotes a general symbol representing each of all other phases; *μ* denotes the CBCT image at the phase designated by its superscript; *x* denotes the 3D coordinates of the CBCT image; DVF denotes the deformation-vector-field; The ${\left\Vert \ast \right\Vert}_{l_2}^2$ terms in Eqs. 1 and 2 calculate the sum of squared errors between the acquired projections *p* and the DRRs of the deformed image; *E*(∗) denotes a quadratic deformation energy term defined to regularize the DVF smoothness [25]; “ *β* ” balances the data fidelity term and the deformation energy term [24]. In general, the 2D-3D deformation algorithm is optimizing a DVF to deform 3D images based on matching 2D projections, as its name “2D-3D” suggests.

The objective functions of Eqs. 1 and 2 are designed as an inverse-consistent deformable registration to solve a pair of forward and inverse DVFs (DVF^0 → *t*^ and DVF^*t* → 0^). The two objective functions are optimized sequentially and the output of one function is input into the other for cyclic iterations. The conversion between DVF^0 → *t*^ and DVF^*t* → 0^ is achieved through the following inverse-consistent constraint:

3 $$ {\mathrm{DVF}}^{0\to t}\circ {\mathrm{DVF}}^{t\to 0}={\mathrm{DVF}}^{0\to t}\left(x+{\mathrm{DVF}}^{t\to 0}\right)+{\mathrm{DVF}}^{t\to 0}\ (x)=0 $$

4 $$ {\mathrm{DVF}}^{t\to 0}\circ {\mathrm{DVF}}^{0\to t}={\mathrm{DVF}}^{t\to 0}\left(x+{\mathrm{DVF}}^{0\to t}\right)+{\mathrm{DVF}}^{0\to t}\ (x)=0 $$

Based on the DVFs optimized by 2D-3D deformation, the SMEIR algorithm applies motion-compensated reconstruction through a modified simultaneous algebraic reconstruction technique (SART) [42], as shown in Eq. 5:

5 $$ {\mu}_j^{0,\left(k+1\right)}={\mu}_j^{0,(k)}+\uplambda \frac{\sum_{t,n}{\mathrm{DVF}}_{jn}^{t\to 0}{}^{\circ}{\sum}_i\left[{a}_{in}\frac{p_i^t-{\sum}_n{a}_{in}{\mu}_n^{t,(k)}}{\sum_{n=1}^J{a}_{in}}\right]}{\sum_{t,n}{\mathrm{DVF}}_{jn}^{t\to 0}{}^{\circ}{\sum}_i{a}_{in}} $$

In Eq. 5, “ *j* ” and “ *n* ” denote voxels in the mCBCT image (reference phase) and the CBCT images at the other phases, respectively; “ *i* ” denotes the projecting ray at pixel location *i* within each projection; “ *J* ” denotes the total number of voxels intercepted by ray *i*; “ *a* ” denotes the intersection length of projecting ray *i* across each voxel; “ λ ” denotes the relaxation factor for SART; “ *k* ” indicates the iteration number. Compared with the conventional SART, the modified SART applies the voxel-wise deformation field ${DVF}_{jn}^{t\to 0}$ to the correction term of each phase, which aligns these correction terms to the same coordinates as those of the reference image, to contribute them all towards the update of the reference image. Using all available information, the modified SART algorithm allows the reconstruction of a high-quality mCBCT with under-sampling streaking artifacts removed. After SART, we applied total-variation regularization on the motion-compensated image to further reduce the image noises and artifacts to improve its quality. The updated mCBCT, *μ*^0^, is further fed as input into the 2D-3D deformation (Eqs. 1–4) to form an iterative loop until final convergence.

**Fig. 2:**
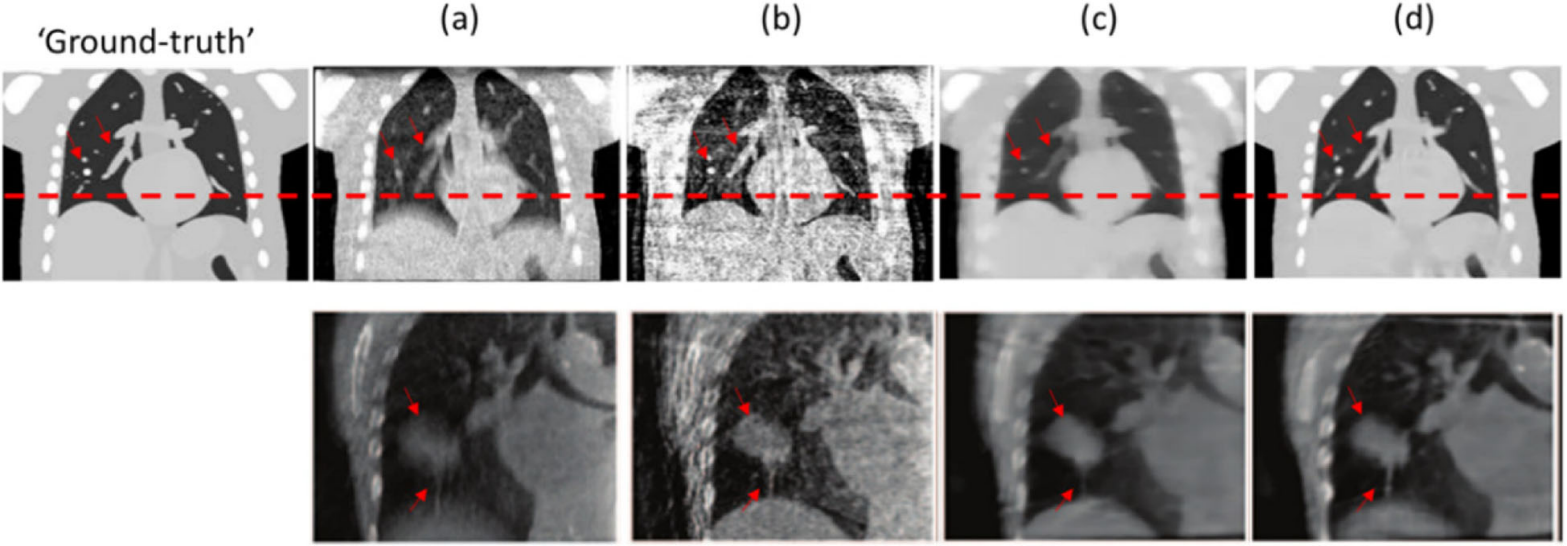
Comparison between the reconstructed images by different methods. **a** Non-phase-sorted FDK images using projections from all respiratory phases; **b** Phase-sorted FDK images using only projections from the peak-expiration phase; **c** Peak-expiration phase image by the algebraic reconstruction technique with total variation-regularization; **d** Peak-expiration phase image by simultaneous motion estimation and image reconstruction. The first row shows the reconstructed images of a digital NCAT phantom [43]. The second row shows the reconstructed images of a real lung cancer patient from clinical projections. The “Ground-truth” image of the NCAT phantom was also presented as a reference. FDK: Feldkamp-Davis-Kress; NCAT: Non-uniform rational B-spline cardiac and torso

Figure 2 compares the reconstructed images by different techniques. The images shown in column (a) were reconstructed by FDK using non-phase-sorted projections from all phases. Though with minimal streaking artifacts, these FDK images presented prominent motion blurriness from mixed-phase reconstruction. By reconstruction using only the phase-sorted projections, the FDK images in column (b) display reduced motion blurriness, but with amplified under-sampling streaking artifacts and noises. With the de-noising effects from total-variation regularization, the images reconstructed by ART-TV substantially reduced the imaging noises and artifacts as compared to (b), however at the cost of missing fine details (as indicated by the arrows). In contrast, the CBCT images reconstructed by the SMEIR algorithm [column (d)] not only removed the noises/artifacts, but the fine details of the images were well preserved through motion-compensated reconstruction with a high-quality motion model.

### The SMEIR-bio algorithm

Though the SMEIR algorithm has proven generally accurate in reconstructing geometrical and intensity information for 4D-CBCT images, some fine anatomical structures, which are small in size, were found not well reconstructed by SMEIR. The organ of lung includes many such structures, including vessels, small bronchioles, and small nodules. Though small in size, these fine structures can serve important landmarks for diagnosis and treatment toxicity evaluation [44]. Accurate reconstruction and presentation of these fine details can be critical to safe and accurate image-guided radiation therapy and adaptive radiation therapy, and pivotal to achieve the maximum therapeutic ratio. These fine details may also convey constructive information towards AI-driven data analysis, such as Radiomics, to predict patient-specific outcomes and treatment responses [45, 46]. It is therefore compelling to correctly reconstruct and display these fine details in lung 4D-CBCTs. However, the 2D-3D deformation technique employed by the original SMEIR algorithm is essentially an intensity-driven approach. The intensity-driven deformable registration techniques may not perform well for these fine structure regions, the deformation of which only lead to small image intensity variations. For 2D-3D deformation, the resulting intensity changes on the 2D cone-beam projections from the deformation of these fine details are even more obscure, leading to minimal changes to the objective functions of 2D-3D deformation (Eqs. 1, 2), especially for under-sampled acquisition scenarios. To improve the motion estimation accuracy of SMEIR of these fine details, we further introduced biomechanical modeling into the SMEIR algorithm (SMEIR-Bio), to solve the DVF via a physics-driven approach [41]. Biomechanical modeling-based deformable registration has been found effective, especially at solving the deformation at low-contrast regions with minimal intensity variation signals [47–51]. By finite element analysis, biomechanical modeling drives the deformation of organs/structures of interest to meet displacement-based or force-based boundary conditions. The elastic properties of these organs/structures are incorporated into the finite element analysis, through a material model, to drive physically-plausible deformation. With minimal dependence on image intensity differences, biomechanical modeling can potentially boost the accuracy of SMEIR in motion estimation and reconstruction of small structures.

**Fig. 3:**
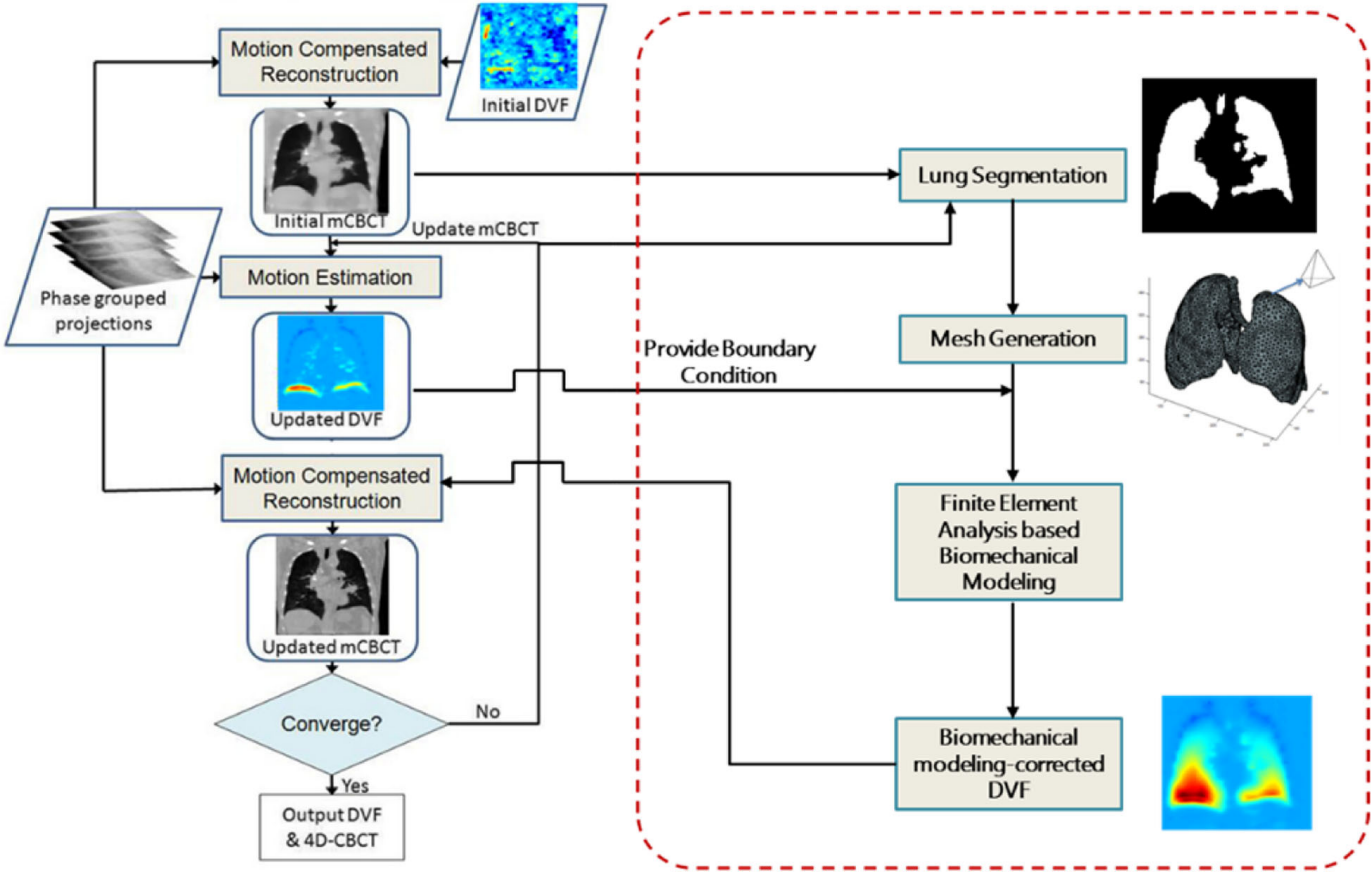
The general flow of the biomechanical modeling-guided SMEIR scheme. 4D-CBCT: 4-Dimensional cone-beam computed tomography; DVF: Deformation-vector-field; mCBCT: Motion-compensated CBCT

The biomechanical modeling process has been incorporated seamlessly into the SMEIR workflow, as illustrated by Fig. 3. To perform biomechanical modeling, we segmented the lung of the mCBCT after motion-compensated reconstruction, and automatically constructed a volumetric tetrahedral mesh using the ISO2MESH and Tetgen packages [52, 53]. The lung was segmented using the automatic “snake” method, which is based on an active contour model [54]. In detail, a few “seeds” were randomly placed within the lung based on intensity-thresholding and grew to fill up the whole lung region, which automatically defined the lung boundary. On the lung boundary, we used the lung surface DVFs solved in the following SMEIR motion estimation (2D-3D deformation) step as displacement-based boundary conditions for biomechanical modeling. With high intensity contrast at the lung boundary, the 2D-3D algorithm could accurately solve lung surface DVFs to provide adequate boundary conditions [27]. With the tetrahedral lung mesh and the boundary conditions, the intra-lung DVFs were deduced via a material model on the relationship between the strain energy and the deformation [55]. Previous studies on lung biomechanical modeling adopted multiple different models, including the linear elastic model [56], the Neo-Hookean model [55], the Ogden model [57], the Marlow model [58], and the Mooney-Rivlin model [27]. Up until now there is no consensus over the most appropriate model, as different models may yield similarly accurate results by using customized material elastic parameters. In our study, we used the Mooney-Rivlin hyper-elastic material, a model usually used to describe soft tissues with relatively large deformation. Detailed information of the model can be found in previous publications [27, 32, 55]. In this study, we modeled the lung as a homogeneous organ with the same elastic parameters throughout the whole volume. Such a strategy has proved effective and efficient by previous studies [27, 47, 55, 56, 58, 59]. After finite element analysis [60], we combined the biomechanical modeling-corrected intra-lung DVFs with outside-lung DVFs, and fed the resulting DVFs into a new motion-compensated reconstruction step before assessing the convergence. If not converged, the mCBCT as well as the inter-phase DVFs would be fed back to initiate a new round of lung segmentation, 2D-3D deformation, biomechanical modeling and motion-compensated reconstruction, until the final convergence was reached.

### The SMEIR algorithm with deep learning-driven DVF fine-tuning (SMEIR-Unet)

**Fig. 4:**
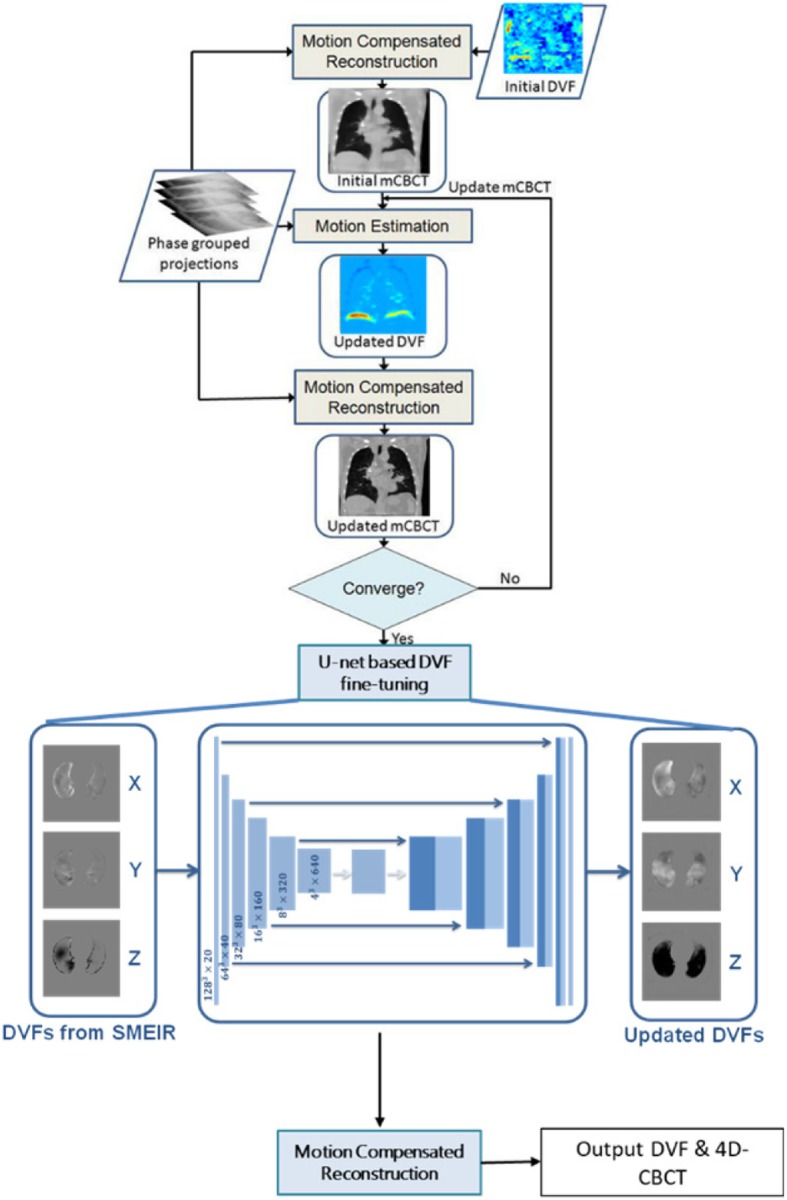
Workflow of the SMEIR-Unet algorithm. Instead of feeding the DVFs into biomechanical modeling, SMEIR-Unet applies a U-net-based convolutional neural network to directly correct the output 2D-3D DVFs to boost their intra-lung accuracy. The corrected DVFs are output as the final inter-phase DVFs, and are applied in a final motion-compensated reconstruction step to generate the mCBCT at the reference phase, as well as the other 4D-CBCT phase images via the inverse DVFs. SMEIR-Unet: Simultaneous motion estimation and image reconstruction with U-net based DVF fine-tuning; DVF: Deformation-vector-field; 4D-CBCT: 4-Dimensional cone-beam computed tomography; mCBCT: Motion-compensated CBCT

The success of SMEIR-Bio requires careful implementation of biomechanical modeling, which involves organ segmentation, tetrahedral mesh generation, material model/parameter assignment and finite element analysis. The additional computational workload and the complexity may reduce the efficiency of 4D-CBCT reconstruction. Recent developments of AI have found many applications in medicine, to potentially improve the accuracy and efficiency over conventional methods [61]. Multiple AI-based studies have been reported in the field of medical image registration [62–65]. Deep convolutional neural networks have been trained to learn DVFs between fixed and moving image pairs to improve deformable registration efficiency. Inspired by the potentials of deep learning, we developed a convolution neural network featuring the U-net structure (SMEIR-Unet) [66], to simplify the SMEIR-Bio workflow and accelerate the computational speed. Instead of applying biomechanical modeling to fine-tune the intra-lung DVFs, we trained a supervised network to establish a direct conversion scheme between the 2D-3D DVFs and the high-quality DVFs. The high-quality DVFs for neural network training were obtained via direct Demons registration between 4D-CT images. In detail, we simulated limited-view phase-binned cone-beam projections from the 4D-CT images and reconstructed 4D-CBCT images and the inter-phase DVFs from the projections using the original SMEIR algorithm. And we used the Demons registration algorithm to register the other phase images of 4D-CT to the reference phase image (motion-compensated phase), to derive the corresponding high-quality Demons DVFs. We paired the 2D-3D DVFs solved by the original SMEIR algorithm with the Demons DVFs, and fed them to train a population-based neural network. To focus the neural network on fine-tuning the intra-lung regions, we cropped both the 2D-3D DVFs and the Demons DVFs using the segmented lung contours at the reference phase. As shown in Fig. 4, the U-net-based convolutional neural network was constructed with two paths: the contraction path and the expansion path. The contraction path contained five blocks, with two convolution layers and a max pooling layer in each block. The expansion path similarly contained six blocks, featuring one deconvolution layer and two convolution layers per block.

For training, validation and testing, we used a 5-fold cross-validation strategy. We grouped the 11 patient-specific 4D-CT sets into 5 groups. And each time we selected 3 groups for training, 1 group for validation and 1 group for testing. The stochastic Adam optimizer is employed to minimizing a cost function defined as the mean squared error between the predicted DVFs and the true Demons DVFs [67]. A parameter-sweeping strategy was employed for learning rate optimization and finalized a learning rate of 5e-5. The batch size was set to 4. The trained U-net could be applied to convert DVFs solved by the SMEIR algorithm, voxel-by-voxel, to improve their accuracy and quality. Note that the SMEIR-Unet algorithm only fine-tunes the DVFs through the trained U-net for once before the final output, in contrast to SMEIR-Bio, which updates the DVFs using biomechanical modeling during every iteration (Figs. 3 and 4).

### Evaluation

For evaluation, we simulated cone-beam projections from high-quality lung 4D-CT images for 4D-CBCT reconstruction, and used the original 4D-CT images as the “Ground-truth” for reference. In this study, we used a dataset including 11 lung patient 4D-CTs acquired on a 16-slice Philips Brilliance CT scanner (Philips Medical Systems, Cleveland, Ohio), which were collected in an academic medical center and not publicly assessable. All the CTs were of the same slice thickness (1.5 mm), while the intra-slice pixel resolutions ranged from 0.78 mm to 0.98 mm, and were uniformly re-sampled to 1.5 mm in our study. Thus all the CTs were of resolution 1.5 mm × 1.5 mm × 1.5 mm. The CTs were of slice dimension 512 × 512, with the slice number ranging from 190 to 270. We simulated 40 projections for each 4D-CT phase, with projection angles evenly distributed across a 360° scan angle, using the Siddon’s ray-tracing technique [68]. We reconstructed 4D-CBCT images from these projections via methods including FDK, algebraic reconstruction technique with total-variation regularization (ART-TV) [69], SMEIR, SMEIR-Bio and SMEIR-Unet for comparison.

To quantitatively assess the accuracy of the motion model (DVFs) solved by different methods, ~ 80 lung landmarks were manually identified by expert radiation oncologists on each phase of the 4D-CT images used in our study. The 3D location changes of the same landmark between different phases were used as the “gold-standard” DVF to calculate the DVF errors of different methods. We evaluated the DVF errors along each of the three Cartesian directions (X, Y, Z), and also in terms of the error vector length.

## Results

**Fig. 5:**
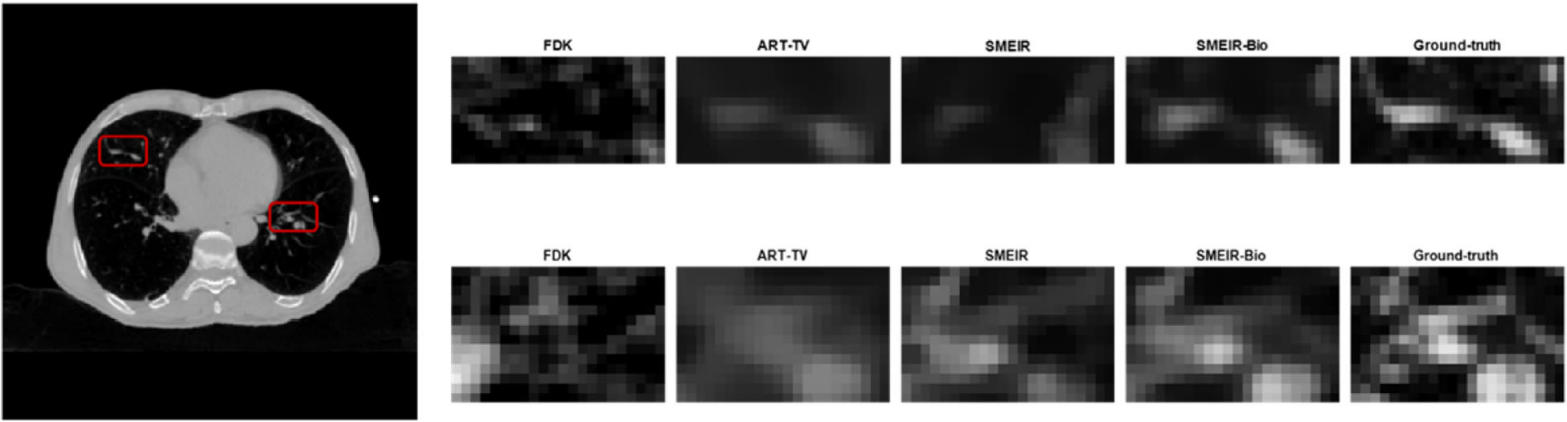
Comparison between the fine details in the CBCT images reconstructed by the FDK, ART-TV, SMEIR and SMEIR-Bio techniques, by using the “Ground-truth” 4D-CT reference phase image for evaluation. All CBCT reconstructions used 40 projections per phase, which were simulated from the “Ground-truth” images. FDK: Feldkamp-Davis-Kress; ART-TV: Algebraic reconstruction technique with total variation-regularization; SMEIR: Simultaneous motion estimation and image reconstruction; CBCT: Cone-beam computed tomography; SMEIR-Bio: Biomechanical modeling-guided SMEIR

Figure 5 compares the small lung details reconstructed by different methods at the reference phase. It can be observed that the FDK image contained structures non-existent on the “Ground-truth” image. These structures were from the under-sampling artifacts and noises. For the ART-TV images, the fine details were over-smoothed and barely recognizable. The original SMEIR algorithm was similarly susceptible to the mis-match of the fine-detail regions from those of the “Ground-truth”, indicating incorrect inter-phase DVFs. In contrast, the SMEIR-Bio algorithm reconstructed small lung details to best match the “Ground-truth”, due to the efficacy of biomechanical modeling in correcting DVFs at these fine-detail regions.

**Fig. 6:**
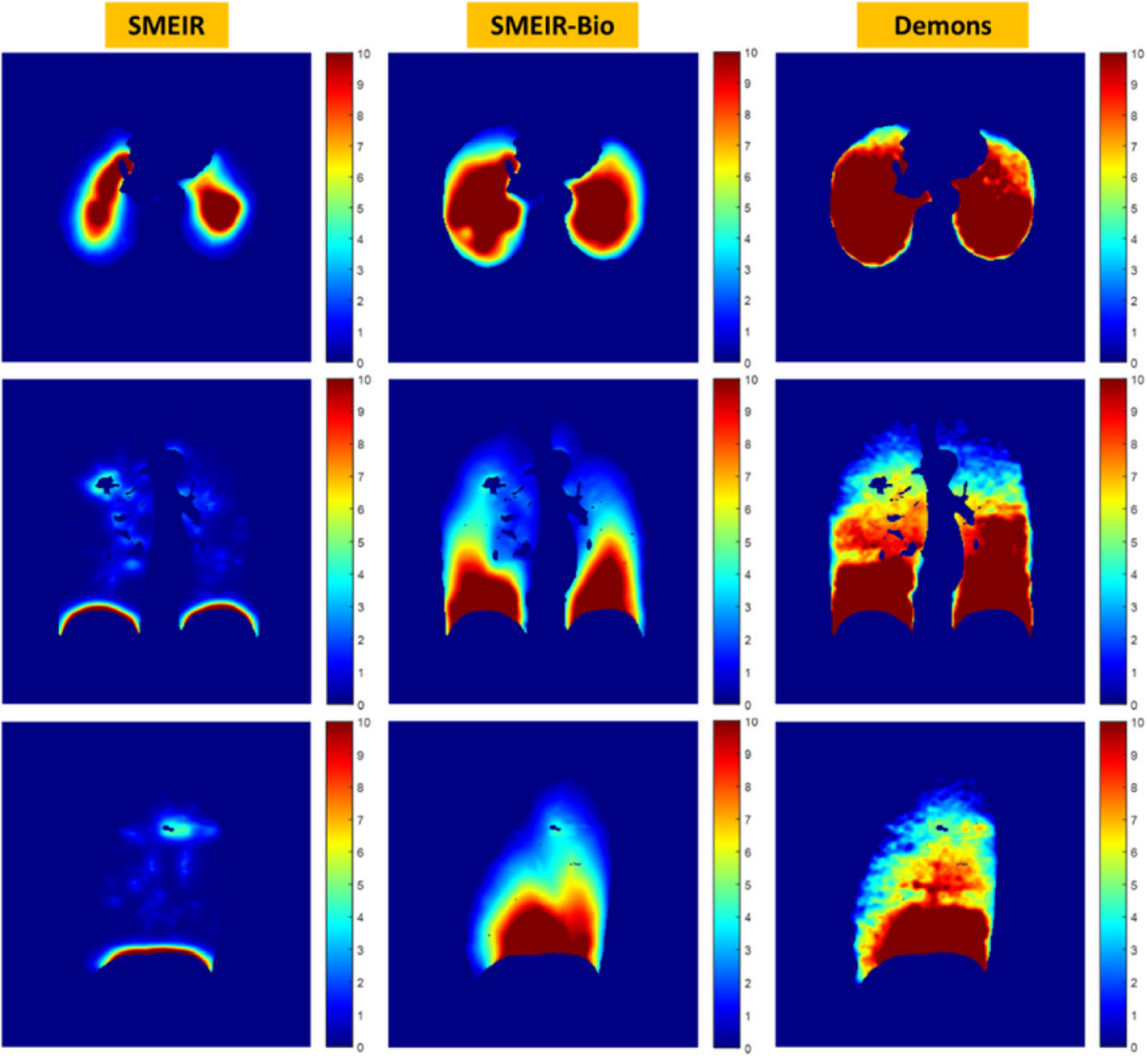
Comparison of DVFs solved by SMEIR, SMEIR-Bio and the Demons registration. The Demons registration was directly performed between the “Ground-truth” high-quality 4D-CT images. SMEIR: Simultaneous motion estimation and image reconstruction; SMEIR-Bio: Biomechanical modeling-guided SMEIR; DVF: Deformation-vector-field

To further evaluate the accuracy of the solved inter-phase DVFs, we used the Demons algorithm to register between the reference phase and the other phases of 4D-CT to generate Demons DVFs for comparison [70]. Since the Demons algorithm was applied directly between high-quality 4D-CT images, the resulting DVFs were of high fidelity to serve as references. Figure 6 shows a comparison between the SMEIR DVF, the SMEIR-Bio DVF and the Demons DVF of three different views. It can be observed that the SMEIR DVF only displayed substantial deformation around the high contrast lung surface. Due to the lack of sufficient intensity variations inside the lung, the intra-lung DVF was not correctly derived by the original SMEIR algorithm. After incorporating biomechanical modeling, SMEIR-Bio substantially improved the DVF accuracy to match with the Demons DVF. In our study, the SMEIR-Bio DVF appeared smoother than the Demons DVF, since we modeled the lung as a homogeneous organ. Introducing heterogeneity during the construction of the biomechanical model may help to further improve the SMEIR-Bio DVF accuracy [51, 71], to potentially better match with the Demons DVF.

**Fig. 7:**
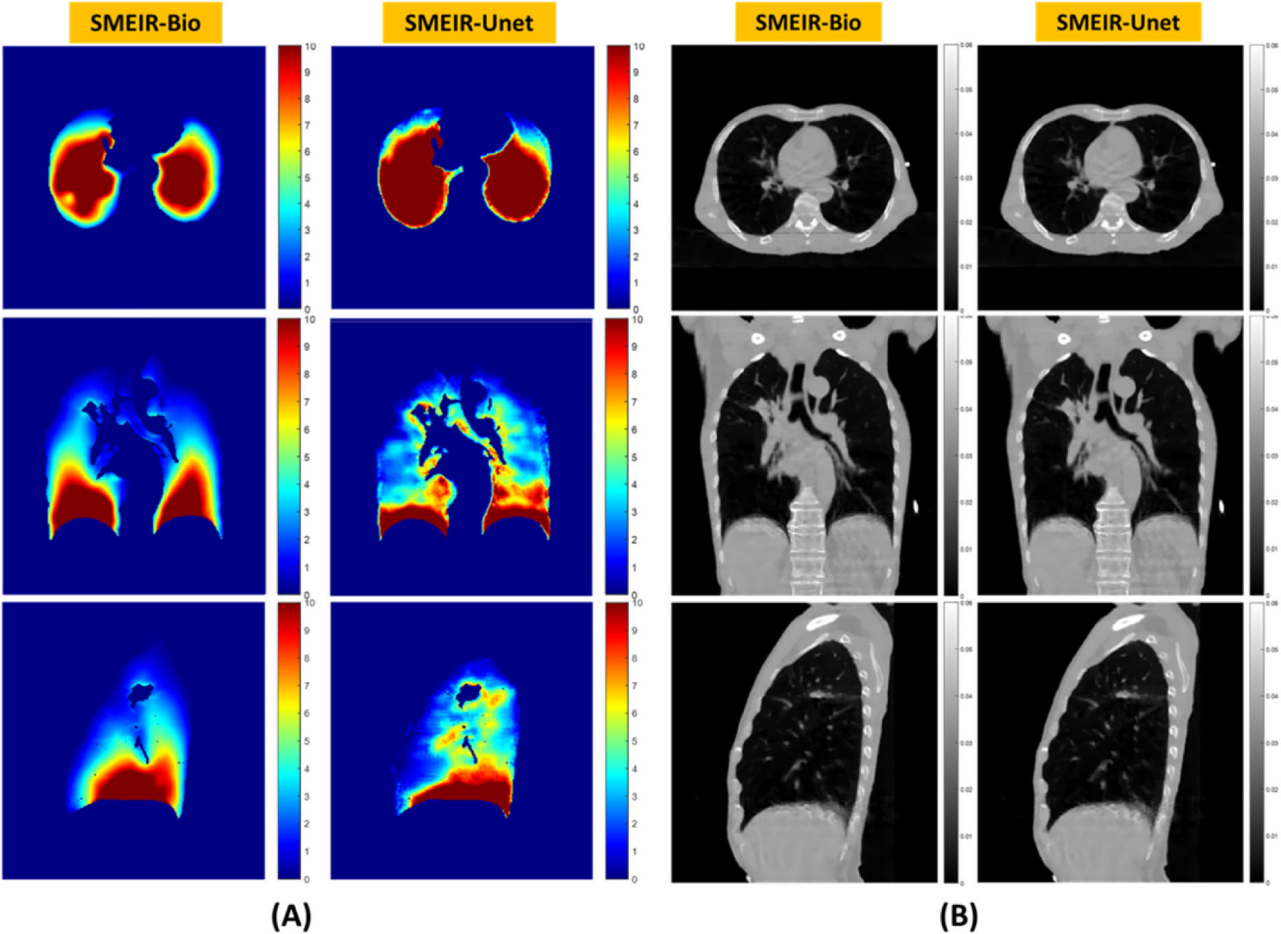
Comparison of DVFs solved by the SMEIR-Bio and the SMEIR-Unet techniques (**a**); Comparison of the corresponding motion-compensated CBCT images reconstructed by the SMEIR-Bio and the SMEIR-Unet techniques (**b**). The DVFs were from the same patient case as Fig. 6, and shown at the same slice locations. SMEIR: Simultaneous motion estimation and image reconstruction; CBCT: Cone-beam computed tomography; SMEIR-Bio: Biomechanical modeling-guided SMEIR; SMEIR-Unet: SMEIR with U-net based DVF fine-tuning; DVF: Deformation-vector-field

As demonstrated in Fig. 7a, the SMEIR-Bio DVFs closely matched the SMEIR-Unet DVFs, and both were substantially improved compared to the original SMEIR DVFs (Fig. 6) in terms of the resemblance to the high-quality Demons DVFs. Due to the homogeneous material modeling, the SMEIR-Bio DVFs were smooth with more gradual spatial variations. In contrast, the SMEIR-Unet DVFs were more heterogeneous, since they were directly learned from the heterogeneous Demons DVFs and inherited similar features. However, a comparison between the reconstructed CBCT images (Fig. 7b) shows the similarity between the reconstructed CBCT images by SMEIR-Bio and SMEIR-Unet, demonstrating that both methods were capable of reconstructing accurate and high-quality CBCT images. We also computed two image quality metrics, the RMSE [39] and the UQI [33], to compare SMEIR-Bio and SMEIR-Unet to the original SMEIR algorithm, based on 10 lung region-of-interests (ROIs) focusing on fine details. For SMEIR-Bio, the average RMSE of the 10 evaluated ROIs was 0.0033 and the average UQI was 0.87. The corresponding results were 0.0035 and 0.93 for SMEIR-Unet, and 0.0039 and 0.67 for the original SMEIR method. Both the SMEIR-Bio and SMEIR-Unet methods improved the accuracy of fine detail reconstruction in lung.

Table 1 shows the quantitative DVF errors for the SMEIR, SMEIR-Bio and SMEIR-Unet methods. Without DVF fine-tuning from biomechanical modeling or convolutional neural network-based conversion, the SMEIR algorithm suffered from the largest DVF error, with the mean vector DVF error around 5 mm. The SMEIR-Bio and SMEIR-Unet techniques achieved appreciable reductions of the DVF errors, with the mean vector DVF error < 3.5 mm for both methods. Compared with SMEIR-Bio, which took > 5 min in building and solving the biomechanical model (including mesh generation, boundary condition assignment, finite element analysis, etc.), SMEIR-Unet only took ~ 10 s to update and fine-tune the DVF for each phase.

**Table 1:** Comparison of DVF errors between SMEIR, SMEIR-Bio and SMEIR-Unet techniques

(Mean ± SD) DVF Error (mm)	SMEIR	SMEIR-Bio	SMEIR-Unet
X	0.79 ± 0.89	0.57 ± 0.78	1.00 ± 0.98
Y	1.55 ± 0.77	0.86 ± 0.62	0.61 ± 0.82
Z	4.31 ± 1.07	2.53 ± 1.00	2.77 ± 0.99
Vector	4.98 ± 1.11	3.11 ± 1.03	3.30 ± 1.08

X, Y, and Z indicate the DVF errors along the three Cartesian directions in space, respectively. The fifth row shows the vector length of the DVF error.* SMEIR* Simultaneous motion estimation and image reconstruction; *DVF* Deformation-vector-field; *SMEIR-Bio* Biomechanical modeling-guided SMEIR; *SMEIR-Unet* SMEIR with U-net based DVF fine-tuning.

## Discussion

4D-CBCT imaging has many potential applications and benefits in radiation therapy. One major application is in image-guided radiation therapy to allow precise cancer treatment. Studies have found that the free-breathing 3D-CBCT imaging may underestimate the motion range of tumors, which can potentially affect the tumor targeting accuracy [72]. The capture of on-board 4D tumor motion by 4D-CBCT will enable better alignment between 4D-CTs and 4D-CBCTs to achieve more accurate and robust patient setup and tumor targeting [73]. The reconstruction accuracy offered by SMEIR-type algorithms fulfills the clinical needs of accurate 4D tumor localization. The ability of SMEIR to reconstruct high-quality images from few projections also renders 4D-CBCT more time-efficient and safe. In comparison to the original SMEIR algorithm, our newly-developed SMEIR-Bio algorithm substantially improved the accuracy of intra-lung DVFs solved using limited-view projections. The derivation of a physically-plausible DVF, from displacement-based boundary conditions, allows us to capture the motion of small, intricate structures within the lung. The biomechanical modeling step requires the input of a lung contour, which was automatically-segmented using the “snake” method in this study. An investigation comparing different lung segmentation methods towards the SMEIR-Bio accuracy was not included in this article. However, since the lung is of high contrast at its boundaries, auto-segmentation is relatively easy and straightforward, and variations among different methods will be small. Furthermore, since DVFs are mostly piecewise constant and smooth, small variations between the lung boundaries segmented by different methods will bear minimal impacts on the extracted boundary condition and the corresponding biomechanical modeling results. In comparison to SMEIR-Bio, which involved the complex biomechanical modeling process, SMEIR-Unet provided similar 4D-CBCT reconstruction and DVF accuracy while with reduced computational workload and improved efficiency.

**Fig. 8:**
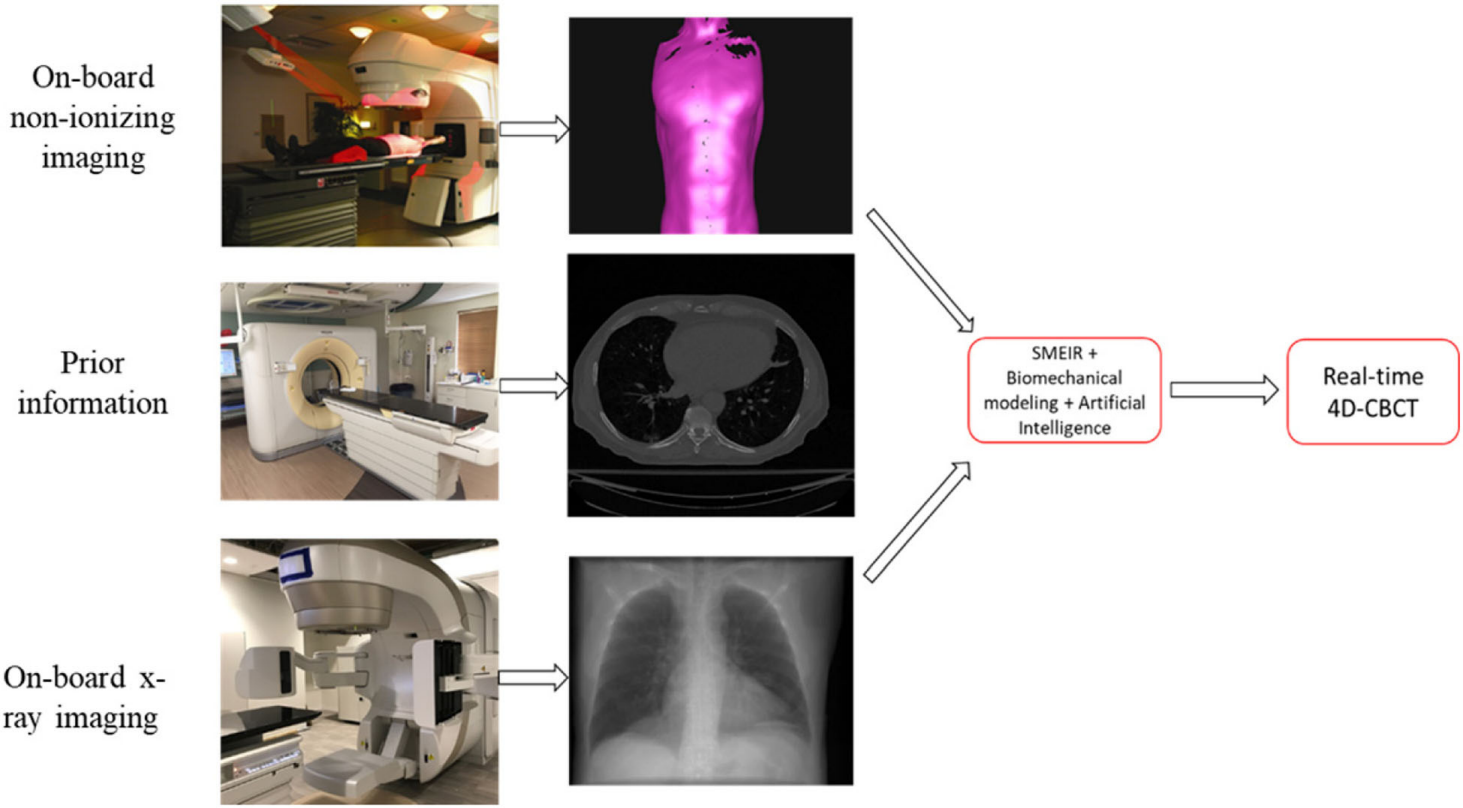
The envisioned future developments towards real-time 4D-CBCT imaging, by combining real-time on-board information provided by non-ionizing imaging signals including the optical surface imaging, and prior information (prior CT/CBCT, prior motion model), and real-time on-board x-ray imaging. Through combining all these information sources, a reconstruction system comprising SMEIR, biomechanical modeling, and AI could potentially achieve real-time imaging to guide the most accurate treatment delivery, and allow on-the-fly plan adjustment to fit the patients’ daily variations. 4D-CBCT: 4-Dimensional cone-beam computed tomography; SMEIR: Simultaneous motion estimation and image reconstruction; AI: Artificial intelligence

Currently, CBCT imaging is mostly applied at the beginning of each radiation therapy fraction, prior to radiation starts. Acquiring more intra-treatment 4D-CBCT images, either between consecutive radiation beams or within each beam [74], will enable continuous updates of tumor motion information to allow treatment adjustments on-the-fly. The ability to closely monitor the motion of moving targets can also promote safety margin reduction to spare more normal structures from being damaged by radiation beams [75]. Our recent developments towards SMEIR acceleration make it feasible to acquire and reconstruct the 4D-CBCT images for treatment guidance in a clinically-acceptable time frame [40], to allow multiple acquisitions and reconstructions during the treatment for continuous target monitoring. The evaluation of the SMEIR algorithm finds it could reconstruct high-quality 4D-CBCT images using as few as 20 projections per phase [38, 39]. Further imaging time reduction by acquiring fewer projections will help approach the goal of real-time volumetric, 4D imaging. Introducing prior information into the reconstruction, like the previously-acquired CT/CBCT images, or a prior motion model, may help to further lower sampling requirements for even faster imaging [25, 28]. On the other hand, high frame-rate non-ionizing imaging signals, such as the surface-guided optical imaging, have also found their potential in helping to achieve real-time volumetric imaging [76]. We envision the next evolution of real-time 4D-CBCT imaging will maneuver the potential of combining the prior information, on-board x-ray imaging, and on-board non-ionizing imaging sources. Through feeding them into a system comprised of advanced reconstruction techniques like SMEIR, biomechanical modeling and AI, a high-quality real-time volumetric image can potentially be reconstructed to maximize the accuracy of radiotherapy (Fig. 8).

**Fig. 9:**
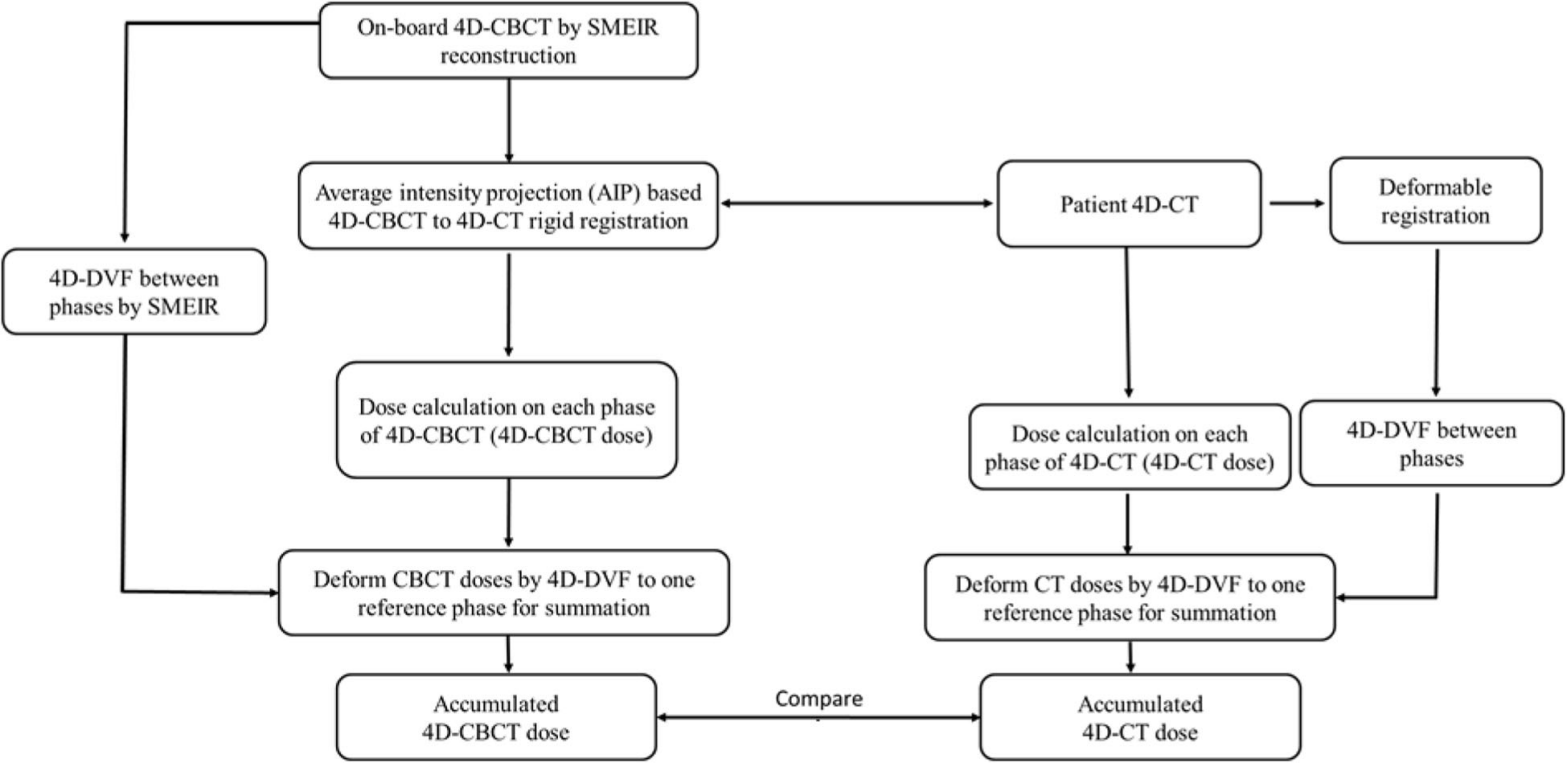
Proposed SMEIR-driven 4D dose accumulation and evaluation workflow for radiation oncology. After applying the rigid registration to align the 4D-CBCT to the 4D-CT coordinates, the 4D-CBCT uses the DVFs solved by SMEIR to deform 4D doses to a single reference phase, in a similar way as the dose accumulation process of 4D-CT. The 4D-CT uses a direct deformable registration algorithm to derive inter-phase DVFs, since the high-quality CT images allow direct inter-phase DVF solution. 4D-CBCT: 4-Dimensional cone-beam computed tomography; SMEIR: Simultaneous motion estimation and image reconstruction; DVF: Deformation-vector-field

In addition to providing anatomical and geometrical information towards more precise radiotherapy targeting, another potential benefit of 4D-CBCT is to calculate delivered radiation doses, and accumulate the delivered doses throughout the treatment course to dynamically assess the need of adaptive radiation therapy, and to provide dosimetric data for dose outcome analysis to fine-tune the treatment prescription [30, 31, 77]. With highly-accurate inter-phase DVFs, the 4D-CBCT solved by SMEIR-type algorithms allows direct and accurate 4D dose accumulation to determine the dose delivered to the gross tumor volumes as well as the normal tissues to evaluate the true dose coverage and the normal tissue toxicity (Fig. 9). By linking 4D-CBCTs acquired at different sessions through inter-session DVFs, we can also accumulate the overall doses delivered during the treatment course for comprehensive treatment evaluation. Currently, one major limitation of applying the CBCTs for direct dose calculation is the inaccurate HU values of the CBCT images, mostly due to the amplified scatter from the cone-beam geometry, as well as the limited field-of-view [78, 79]. Some studies proposed to circumvent the issue through direct CT-CBCT registration to calculate treatment doses on the deformed CT image instead [80, 81]. However, such methods are susceptible to the registration errors between the planning CT and on-board CBCT images. It would be ideal to directly improve the CBCT HU accuracy to allow direct dose calculation on CBCT. Multiple methods have been developed to reduce and correct the scatter signal, including experimental measurements, hardware-based corrections and Monte-Carlo simulations [79, 82, 83]. Introducing such methods into the clinic will allow substantial improvements of the CBCT HU accuracy. On the other hand, the issue of limited field-of-view can be potentially addressed through incorporating prior information into the CBCT reconstruction, which also enjoys the benefit of introducing more accurate HUs [30]. Recent developments of AI-based techniques were found effective to convert the CBCT images directly to improve their quality to match that of planning CT images for accurate dose calculation [84]. Such techniques could be grafted with SMEIR-type algorithms to yield the final 4D-CBCT directly applicable for accurate dose calculation. Based on the DVF-accumulated doses on each treatment fraction and throughout all fractions, and based on the DVF-propagated tumor and normal tissue contours [32, 85], decisions can be rendered in regards to the necessity of adaptive radiation therapy. Based on high-quality CBCTs, radiotherapy plans can be adapted and optimized to meet the patient’s clinical needs to maximize the radiotherapy benefits.

## Conclusions

The fast development of medical imaging has allowed more precise radiation therapy treatments and is continuously pushing the boundary of technology towards safer beam delivery and more effective normal tissue sparing. 4D-CBCT plays a key role in managing sites involving moving targets such as lungs and livers. Through combining motion estimation and motion-compensated reconstruction, the SMEIR algorithm allows low-dose and faster 4D-CBCT imaging to better meet the clinical demands. The introduction of biomechanical modeling and U-net based DVF fine-tuning further develops the original SMEIR algorithm to better reconstruct small, fine details in addition to bulky structures. Through introducing more information, including the prior information and the high frame-rate, non-ionizing imaging technique, we could potentially accelerate the 4D-CBCT acquisition to allow near real-time imaging to achieve the most precise tumor targeting with a minimal safety margin. In addition to image-guidance, the 4D-CBCT and the inter-phase DVFs solved by SMEIR-type algorithms also allow dose calculation and accumulation to guide intra-course adaptive radiation therapy to tailor the plan to better deliver patient-specific cancer care, and provides abundant information to assess the dose-treatment outcomes, on both tumor control and normal tissue toxicity.

## Data Availability

Please contact the corresponding author for data requests.

## Abbreviations

4D-CBCT: 4-dimensional cone-beam computed tomography
AI: Artificial intelligence
ART-TV: Algebraic reconstruction technique with total variation-regularization
DRRs: Digitally-reconstructed-radiographs
DVF: Deformation-vector-field
FDK: Feldkamp-Davis-Kress
mCBCT: Motion-compensated CBCT
NCAT: Non-uniform rational B-spline cardiac and torso
RMSE: Root-mean-squared-error
ROIs: Region-of-interests
SART: Simultaneous algebraic reconstruction technique
SMEIR: Simultaneous motion estimation and image reconstruction
SMEIR-Bio: Biomechanical modeling-guided SMEIR
SMEIR-Unet: SMEIR with U-net based DVF fine-tuning
UQI: Universal quality index
